# Bitter tastants and artificial sweeteners activate a subset of epithelial cells in acute tissue slices of the rat trachea

**DOI:** 10.1038/s41598-019-45456-w

**Published:** 2019-06-20

**Authors:** Chiara Lasconi, Simone Pifferi, Andres Hernandez-Clavijo, Flavia Merigo, Maria Paola Cecchini, Kevin Y. Gonzalez-Velandia, Emilio Agostinelli, Andrea Sbarbati, Anna Menini

**Affiliations:** 1 Department of Neurosciences, Biomedicine and Movement Sciences, Anatomy and Histology Section, University of Verona, School of Medicine, Verona, Italy; 2 Neurobiology Group, SISSA, International School for Advanced Studies, Trieste, Italy

**Keywords:** Genetics, Physiology

## Abstract

Bitter and sweet receptors (T2Rs and T1Rs) are expressed in many extra-oral tissues including upper and lower airways. To investigate if bitter tastants and artificial sweeteners could activate physiological responses in tracheal epithelial cells we performed confocal Ca^2+^ imaging recordings on acute tracheal slices. We stimulated the cells with denatonium benzoate, a T2R agonist, and with the artificial sweeteners sucralose, saccharin and acesulfame-K. To test cell viability we measured responses to ATP. We found that 39% of the epithelial cells responding to ATP also responded to bitter stimulation with denatonium benzoate. Moreover, artificial sweeteners activated different percentages of the cells, ranging from 5% for sucralose to 26% for saccharin, and 27% for acesulfame-K. By using carbenoxolone, a gap junction blocker, we excluded that responses were mainly mediated by Ca^2+^ waves through cell-to-cell junctions. Pharmacological experiments showed that both denatonium and artificial sweeteners induced a PLC-mediated release of Ca^2+^ from internal stores. In addition, bitter tastants and artificial sweeteners activated a partially overlapping subpopulation of tracheal epithelial cells. Our results provide new evidence that a subset of ATP-responsive tracheal epithelial cells from rat are activated by both bitter tastants and artificial sweeteners.

## Introduction

Vertebrate taste receptors were first identified in taste receptor cells located in the oral cavity, where they detect ligands for taste modalities such as sweet, bitter, umami, sour and salty. The ligand-receptor binding activates a transduction cascade leading to a signal that is transmitted to the brain and produces a taste sensation^1^. In addition, bitter and sweet receptors were also detected in several extra-oral systems including airways, stomach, intestine, pancreas, thyroid and brain, although here their physiological functions are still largely unknown^2–5^.

Bitter and sweet receptors are G–protein coupled receptors. Bitter receptors belong to the taste receptor family 2 (T2Rs), composed of about 30 members^6–8^, while sweet receptors belong to the taste receptor family 1 (T1Rs) composed by three different members (T1R1, T1R2, T1R3). These latter can combine forming the umami taste receptor (T1R1-T1R3), the heterodimeric sweet taste receptor (T1R2-T1R3) and the homodimeric low-affinity sweet taste receptor (T1R3-T1R3)^9,10^. Bitter and sweet receptors are G–protein coupled receptors, composed of a GTP-binding α subunit and βγ-subunit^11^. The G protein *α*-subunit (*α*-gustducin) has been identified and cloned from taste tissue, and it is expressed in taste buds of all taste papillae (circumvallate, foliate and fungiform)^12^. The binding of ligands to bitter and sweet taste receptors activates at least one common signaling pathway leading to the dissociation of a heterotrimeric G-protein into α and βγ subunits. The latter activates phospholipase-C-beta-2 (PLCβ2)^13^, mediating the synthesis of inositol-3-phosphate (IP_3_), which gates IP_3_ receptor type 3 (IP3R3) on the endoplasmic reticulum, causing Ca^2+^ release in the cytosol^13–15^. The increased intracellular Ca^2+^ concentration activates two members of the transient receptor potential cation channel subfamily M, TRPM5 and TRPM4, causing membrane depolarization, action potential generation and release of ATP through CALHM1-CALHM3 channels^13,16–21^.

Several elements of the taste transduction-signaling pathway such as α-gustducin, PLCβ2, IP3R3 and TRPM5 have been found in various extra-oral tissues (for review see^4,22^). The presence of similar pathways in different cell types suggests the existence of conserved mechanisms in a wide range of tissues and highlights that receptors involved in the oral gustatory pathway have other interesting and additional roles in extra-oral organs^23–25^.

Interestingly, bitter and sweet receptors and some elements of the taste transduction cascade are expressed in several types of cells lining the lumen of the airway epithelium^2,22^. Morphologically, the airway epithelium is mainly composed of ciliated cells, non-ciliated mucus secretory cells (goblet cells), and basal cells. In addition, a chemosensory population, generally named solitary chemosensory cells (SCCs), has been described. It is composed of different cell types, not aggregated in buds, but scattered inside the epithelium, including neuroendocrine, brush, and microvillar cells^2,22,25–27^.

In rodents, airway SCCs express T2Rs and T1R3 receptors and components of the sweet and bitter transduction pathway such as α-gustducin, PLCβ2 and TRPM5^28–30^. In addition, it was demonstrated that some airway chemosensory cells also express cholinergic traits and lie close to, or contact, subepithelial nerves expressing nicotinic acetylcholine receptors^31–34^.

Ciliated cells bear several motile cilia protruding from the apical surface to the lumen. Traditionally, motile cilia serve principally a mechanical function while immotile cilia are generally considered to be sensory. Motile cilia are microtubule-based organelles that beat in highly regulated and synchronized way generating metachronal waves responsible for moving the mucus from the lower airways to the upper airways^35,36^. The ciliary beat frequency and therefore the mucociliary clearance is modulated in response to both physical (pH, force, temperature) and chemical stimuli and alteration of ciliary mobility is involved in many diseases^35,36^. Shah *et al*.^37^ showed that some bitter tastants, such as denatonium benzoate and quinine, elicit an increase both of intracellular Ca^2+^ concentration and of the ciliary beat frequency in ciliated cells from cultures obtained from human trachea and bronchi explants. In addition, they demonstrated that ciliated cells express several T2R bitter receptors and some elements of the taste transduction cascade. T2R bitter receptors were subsequently found also in ciliated cells of the human sinonasal epithelium, where stimulation with bitter tastants increased the ciliary beat frequency, raising the mucociliary transport rate^38–40^.

We found that rat tracheal ciliated cells express the T1R3 receptor^41^. In particular, T1R3 localizes on the apical surface beneath the cilia, in some spots along the cilia and on the basolateral membrane. The localization of the T1R3 allows a direct interaction with the luminal content and it resembles the pattern of T2R in ciliated cells from cultures of human trachea^37,41^. In addition, the glucose transporter GLUT2 colocalizes with T1R3 on the apical membrane of some ciliated cells, suggesting a possible role for glucose sensing regulation mediated by ciliated cells^41^.

On the basis of these previous findings, we sought to investigate the responses of rat tracheal epithelial cells to both artificial sweeteners and bitter tastants. For this purpose, we developed a preparation of rat acute tracheal slices and we measured the responses with confocal Ca^2+^ imaging. The use of tracheal acute slices has the advantage of preserving the native tissue organization while avoiding possible artifacts due to cell culturing. We found that rat tracheal epithelial cells are a heterogeneous population able to respond to different combinations of bitter tastants and artificial sweeteners.

## Results

### Ca^2+^ imaging recordings from acute slices of the trachea

**Figure 1:**
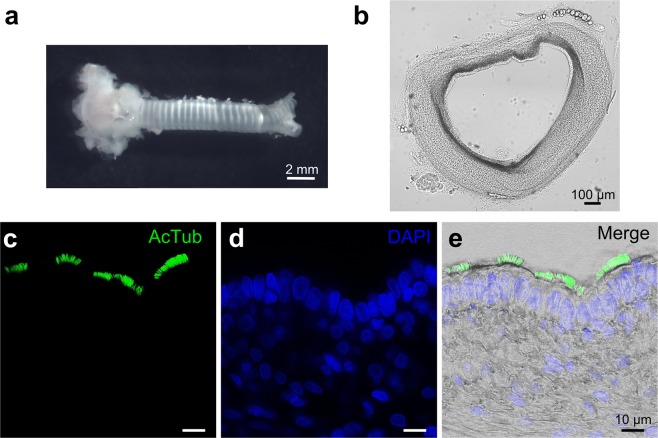
Preparation of rat acute tracheal slices. (**a**) Photomicrograph of the trachea freshly extracted from a newborn rat. (**b**) 200 μm thick coronal slice with preserved morphology. (**c**) A tracheal slice was immunolabeled with anti-acetylated α-Tubulin antibody (AcTub) to visualize the ciliated cells. (**d**) Nuclei were stained with DAPI. (**e**) Digital addition of fluorescence and bright-field images.

We developed a preparation of acute tracheal slices from neonatal rats (Fig. 1a,b) and used these slices to measure responsiveness of individual cells to various tastants with the Ca^2+^ imaging technique. The major advantage of using acute tracheal slices instead of cultures of the tracheal epithelium consists in the maintenance of the native organization. Indeed, Fig. 1c–e shows immunohistochemical analysis from the membranous region of an acute slice of trachea. The slice preserved the cross-sectional structure and individual cells could be distinguished by their morphology. Several cilia protruding from apical membranes towards the lumen were stained with acetylated tubulin, a specific marker for cilia.

We performed Ca^2+^ imaging in acute slices from the membranous region of the trachea and observed that numerous cells facing the lumen had beating cilia (Supplementary Video S1). As previous data have shown that tracheal ciliated cells from various species respond to ATP stimulation with an increase in intracellular Ca^2+^ concentration^42–44^, we used the responsiveness to ATP as control of cell viability. The supplementary video S1 shows a significant fluorescence change in response to ATP in several cells. The arrows indicate two ciliated cells in which fluorescence changes were also detected in the cilia, although it may be difficult to see changes in the cilia in other cells. Due to this technical limitation, we will simply refer to them as tracheal epithelial cells.

**Figure 2:**
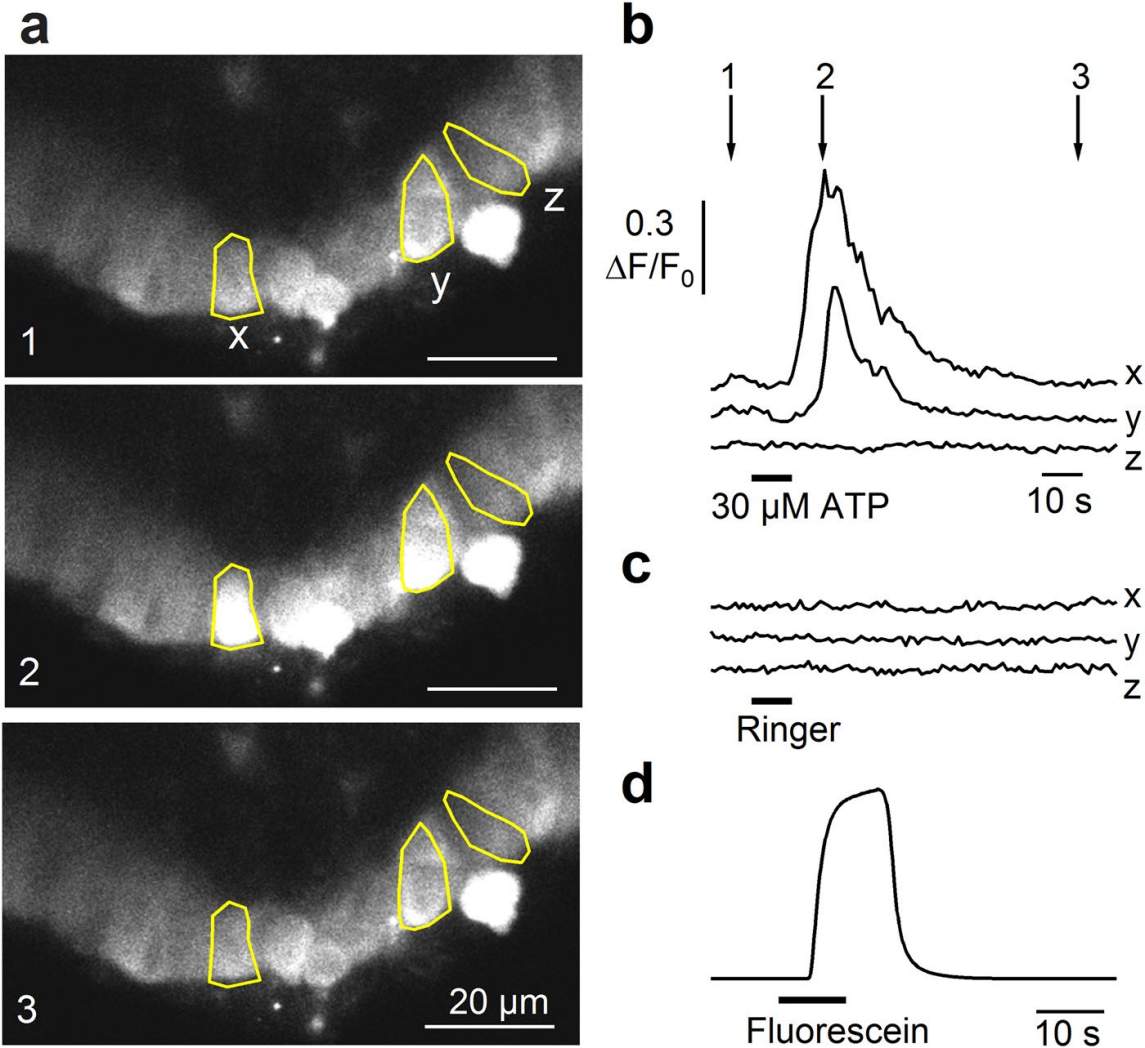
Responses of tracheal epithelial cells to ATP. (**a**) Representative sequences of confocal images from Cal520-AM loaded tracheal slice before (1), at the peak (2), and after (3) the response activated by 30 μM ATP for 10 s. (**b**) Calcium transients recorded in the cells highlighted in panel a. Time points indicated by arrows correspond to frame numbers in a. (**c**) In the same cells, the application of Ringer’s solution did not evoke significant calcium signals. (**d**) Fluorescence evoked by application of fluorescein was used to measure the rate of perfusion system.

Figure 2a shows three confocal fluorescence images from a rat tracheal slice before (1), during (2), and after (3) the response to 30 μM ATP. We selected three cells (x, y, z) and calculated the normalized fluorescence changes (ΔF/F_0_). Figure 2b shows that ATP induced a significant transient increase of the intracellular Ca^2+^ concentration in cells x and y that returned to baseline level after several seconds, while ATP did not induce any Ca^2+^ change in cell z. The change of intracellular Ca^2+^ was measured in the soma. In each cell, as control, we also perfused Ringer’s solution to verify possible artifacts due to slice movements during the solution switching and discarded from the analysis all the cells showing a change of Cal-520 signal during Ringer application (Fig. 2c). We measured the time necessary for stimulus arrival at the tracheal slice from our perfusion system by adding fluorescein to Ringer’s solution and estimated a delay of about 4 s from valve opening of our perfusion system, and a complete change of solution in about 10 s (Fig. 2d). The longer delay in ATP response (about 10 s in Fig. 2b) suggests that ATP activated a second messenger cascade to produce a transient increase of intracellular Ca^2+^, most likely the activation of G-protein mediated signaling through metabotropic P2Y receptors.

Recordings from several slices showed that at least 70% of the epithelial cells (303 of 430 cells from 20 slices) were viable and responded to ATP stimulation with a transient increase of intracellular Ca^2+^.

### Tracheal epithelial cells respond to bitter tastants and artificial sweeteners

**Figure 3:**
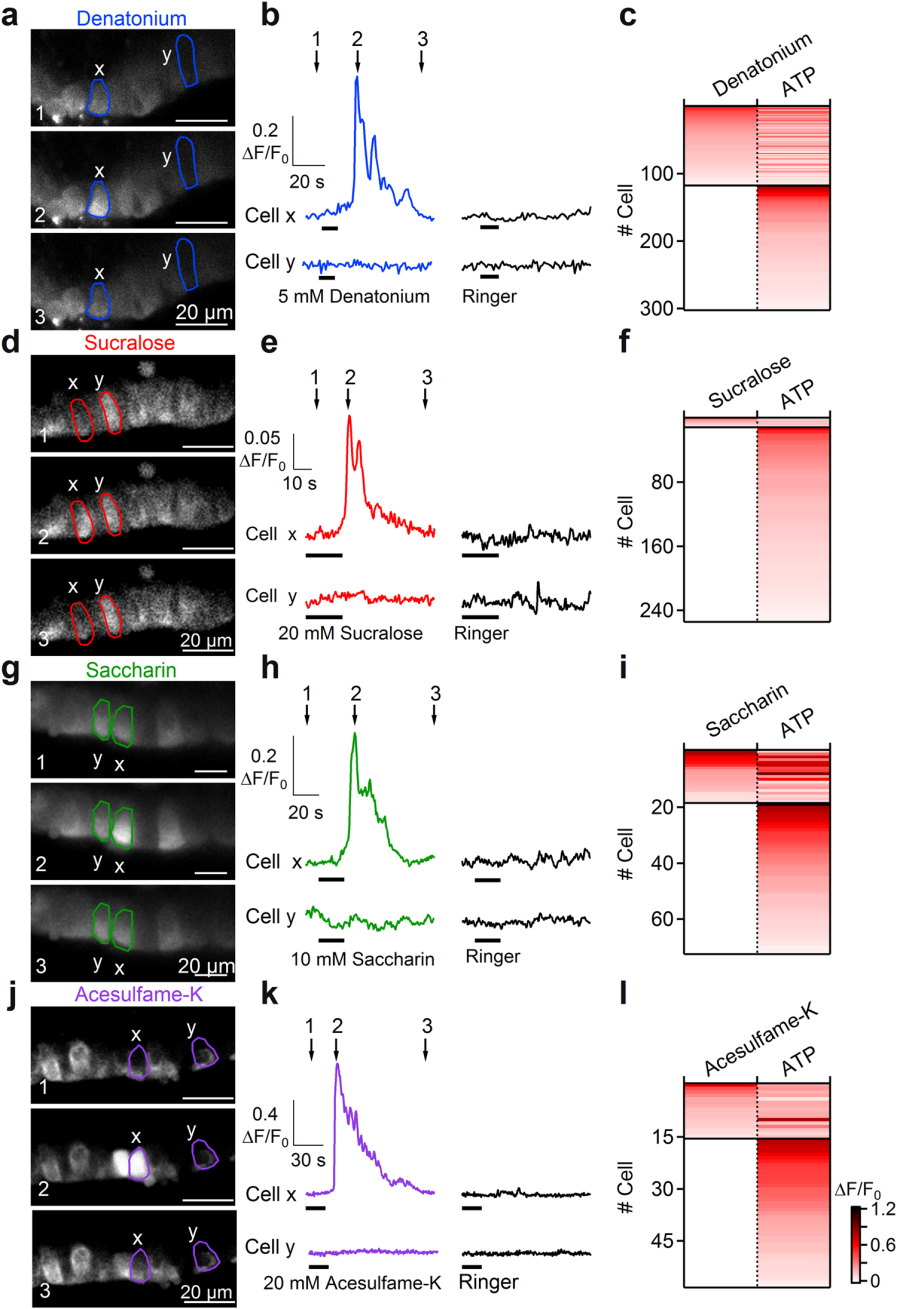
Tracheal epithelial cells respond to bitter tastants and artificial sweeteners. (**a**–**d**–**g**–**j**) Representative sequences of confocal images from Cal520-AM loaded tracheal slices before the response (1), at the peak of calcium response activated by 5 mM denatonium, 20 mM sucralose, 10 mM saccharin or 20 mM acesulfame-K (2), and after the response (3). (**b**–**e**–**h**–**k**) Calcium transients recorded in individual cells (highlighted in **a**–**d**–**g**–**j**) responding to the indicated stimuli. Time points indicated by arrows correspond to frame numbers in a. (**c**–**f**–**i**–**l**) Heat maps of normalized change in fluorescence intensity following stimulation with the indicated compounds. Thin black lines indicate divisions among cells with different response profiles.

Denatonium is a well-known bitter tastant which has been shown to produce an increase in intracellular Ca^2+^ and in the ciliary beat frequency of ciliated cells from cultured human airway epithelia^37^. Figure 3a shows three confocal fluorescence images of a rat tracheal slice before (1), during (2), and after (3) the response to 5 mM denatonium benzoate. Measurements of ΔF/F_0_ in a selected cell (cell x) show that the application of denatonium induced a significant transient increase of the intracellular Ca^2+^ concentration that returned to baseline after some seconds (Fig. 3b upper traces). In the same slice, some cells did not show a significant response to denatonium (cell y, Fig. 3b) but responded to ATP stimulation (Fig. 3c). Recordings from several slices show that 39% of the ATP-responsive cells (118 of 303 cells from 20 slices) responded to denatonium application with a transient increase of intracellular Ca^2+^ (Fig. 3c).

To test sweet tastants, we selected the artificial sweeteners sucralose, saccharin and acesulfame-K. Artificial sweeteners were chosen because it has been shown that a much lower concentration of these substances compared to sugars is usually sufficient to stimulate sweet receptors^9,45^, although it must be noted that saccharin and acesulfame-K (but not sucralose) also activate human bitter receptors^46^. Figure 3d–g–j shows confocal fluorescence images of rat tracheal slices before, during and after stimulation with 20 mM sucralose, 10 mM saccharin, or 20 mM acesulfame-K. Measurements of ΔF/F_0_ in selected cells show that the application of each artificial sweetener induced a significant reversible increase of the intracellular Ca^2+^ concentration in a subset of ATP-responsive tracheal epithelial cells (Fig. 3e–h–k). Recordings from several slices (Fig. 3f–i–l) show a Ca^2+^ signal in response to sucralose in 5% (12 of 255 cells from 17 slices), to saccharin in 26% (19 of 73 cells from 7 slices), and to acesulfame-K in 27% (16 of 59 cells from 8 slices) of the ATP-responsive cells.

**Figure 4:**
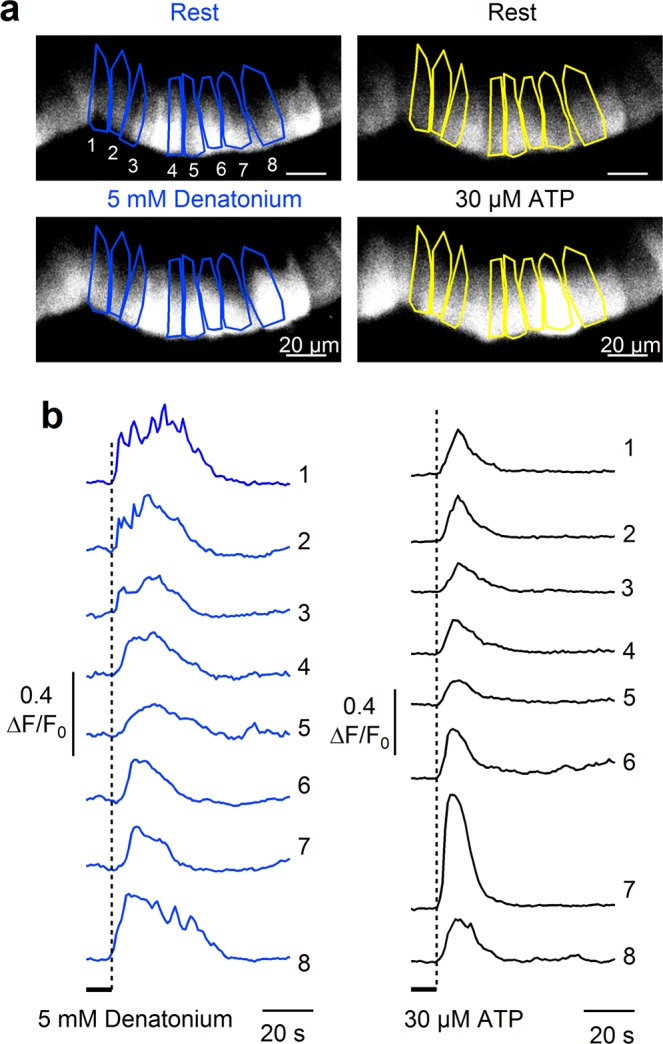
Temporal profiles of tracheal epithelial cell responses to denatonium. (**a**) Representative confocal images from Cal520-AM loaded tracheal slice before (upper panels) and during the stimulation with 5 mM denatonium (left) or 30 μM ATP (right). (**b**) Calcium transients recorded from the cells highlighted in a responding to 5 mM denatonium (left) or to 30 μM ATP (right).

To investigate the temporal profile of responses from tracheal epithelial cells, we selected slices with several responsive cells. Figure 4a shows confocal fluorescence images of a rat tracheal slice before and during stimulation with denatonium or ATP. The delay from the time of valve opening of our perfusion system for stimulus application and the beginning of the responses to denatonium or to ATP for each cell was very similar (Fig. 4b), with an average value of 12.1 ± 0.6 s (n = 8) for denatonium not significantly different from the value of 11.0 ± 0.2 s (n = 8) for ATP suggesting that both compounds activate epithelial cells with a similar time course and confirming previous results in human tracheal and bronchial epithelium cultures^37,47^. Similar results were obtained for the delay of Ca^2+^ signals in response to saccharin, acesulfame-K, sucralose or to ATP.

To investigate whether the Ca^2+^ increase originated in some cells, such as SCCs, and subsequently propagated to the surrounding epithelial cells through gap junctions, we applied the gap junction blocker carbenoxolone, previously used to isolate epithelial cells’ communication in airways^47,48^.

We first performed control experiments to measure the time necessary to obtain gap junctions blockage on a tissue slice preparation with our perfusion system. We used slices of the olfactory epithelium and measured the electrical coupling through gap junctions of supporting cells, that have been previously shown to be sensitive to 100 µM carbenoxolone inhibition^49^. We obtained patch-clamp recordings in the whole-cell configuration from olfactory supporting cells and measured how the input resistance value changed with time upon application of 100 µM carbenoxolone (Supplementary Fig. 1). The input resistance value increased and reached a steady state value in less than 3 minutes after carbenoxolone application, indicating that 3 minutes of blocker application to the olfactory epithelium slice with our perfusion system were sufficient to inhibit the gap junctions between supporting cells (Supplementary Fig. 1).

**Figure 5:**
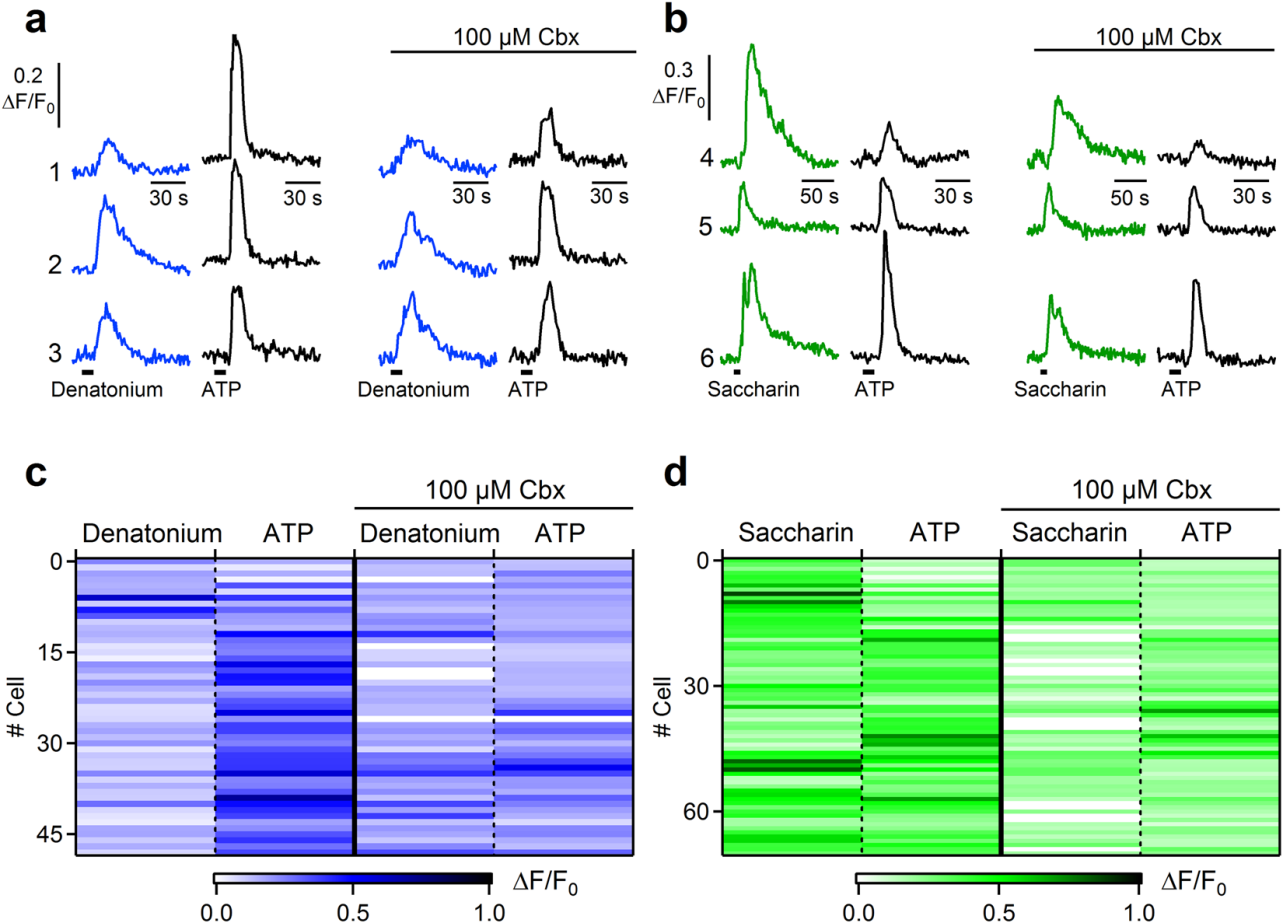
The gap junction blocker carbenoxolone does not affect the frequency of responsive tracheal epithelial cells to denatonium and saccharin. (**a**) Representative calcium transients recorded from three individual tracheal epithelial cells in response to 5 mM denatonium (blue traces) or 30 µM ATP (black traces) before and after application of 100 µM carbenoxolone (Cbx). (**b**) Calcium transients in response to 20 mM saccharin (green traces) or 30 µM ATP (black traces) before and after application of 100 µM carbenoxolone. (**c**,**d**) Heat maps of normalized change in fluorescence intensity following stimulation with the indicated compounds before and after application of 100 µM carbenoxolone.

Then, we tested the effect of carbenoxolone on epithelial cells in tracheal slices. We tested 5 mM denatonium and 30 µM ATP in control conditions and 5 minutes after the application of 100 µM carbenoxolone (Fig. 5a,c). Recordings from several slices showed that up to 90% of the cells (44 of 49 cells from 5 slices) still responded after the gap junction blockage with carbenoxolone (Fig. 5c).We compared these results with a control group of experiments with sequential application of denatonium (in the absence of carbenoxolone, Fig. S2a). Control experiments showed that 85% of the cells responded to the second application of denatonium after the perfusion of Ringer’s solution for 5 minutes (40 of 47 cells from 3 slices). The chi-squared test showed that frequencies of responsive cells in the presence of carbenoxolone or control experiments were not significantly different (χ^2^ = 0.48, p = 0.49).

Very similar results were obtained also when tracheal epithelial cells were stimulated with 10 mM saccharin and 30 µM ATP (Fig. 5b,d), indeed 80% of the cells retained their responsiveness after carbenoxolone application (57 of 71 cells from 5 slices) and control experiments with sequential application of saccharin (without carbenoxolone, Fig. S2b) showed that 92% of the cells responded to the second application of saccharin after Ringer’s solution perfusion (33 of 36 cells from 2 slices). The chi-squared test showed that frequencies of responsive cells in the presence of carbonexolone or control experiments were not significantly different (χ^2^ = 2.31, p = 0.13). These results suggest that responses to denatonium or to saccharin were not mainly mediated by spread of Ca^2+^ signaling through gap junctions.

**Figure 6:**
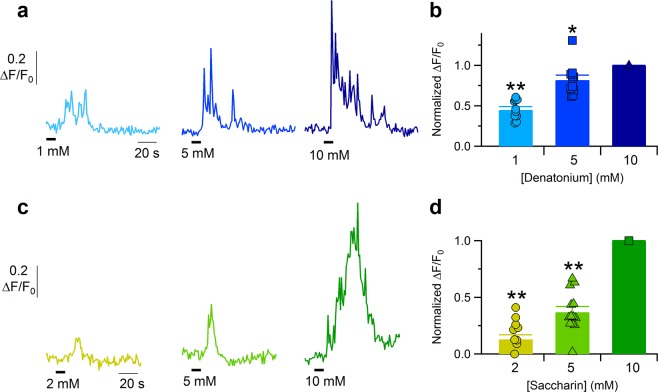
Dose-response of tracheal epithelial cells to denatonium and saccharin. Representative calcium transients recorded from two epithelial cells stimulated with solutions containing different denatonium (**a**) or saccharin (**c**) concentrations. (**b**,**d**) Scatter dot plot and bar plot showing the normalized peak fluorescence intensity plotted versus the increasing agonist concentration. Bar plot shows mean ± sem (*P < 0.05, **P < 0.01 One sample sign test).

We also tested denatonium and saccharin at additional concentrations and found out that responses were concentration-dependent. Denatonium was tested at 1, 5 or 10 mM (Fig. 6a, 10 cells from 3 slices) and the intracellular Ca^2+^ increase in tracheal epithelial cells depended on denatonium concentration. On average, 1 mM evoked about 50% of the response induced by 10 mM denatonium (Fig. 6b). Saccharin was tested at 2, 5 or 10 mM (Fig. 6c, 13 cells from 3 slices) and 5 mM induced on average about 40% of the Ca^2+^ signal activated by 10 mM saccharin (Fig. 6d).

**Figure 7:**
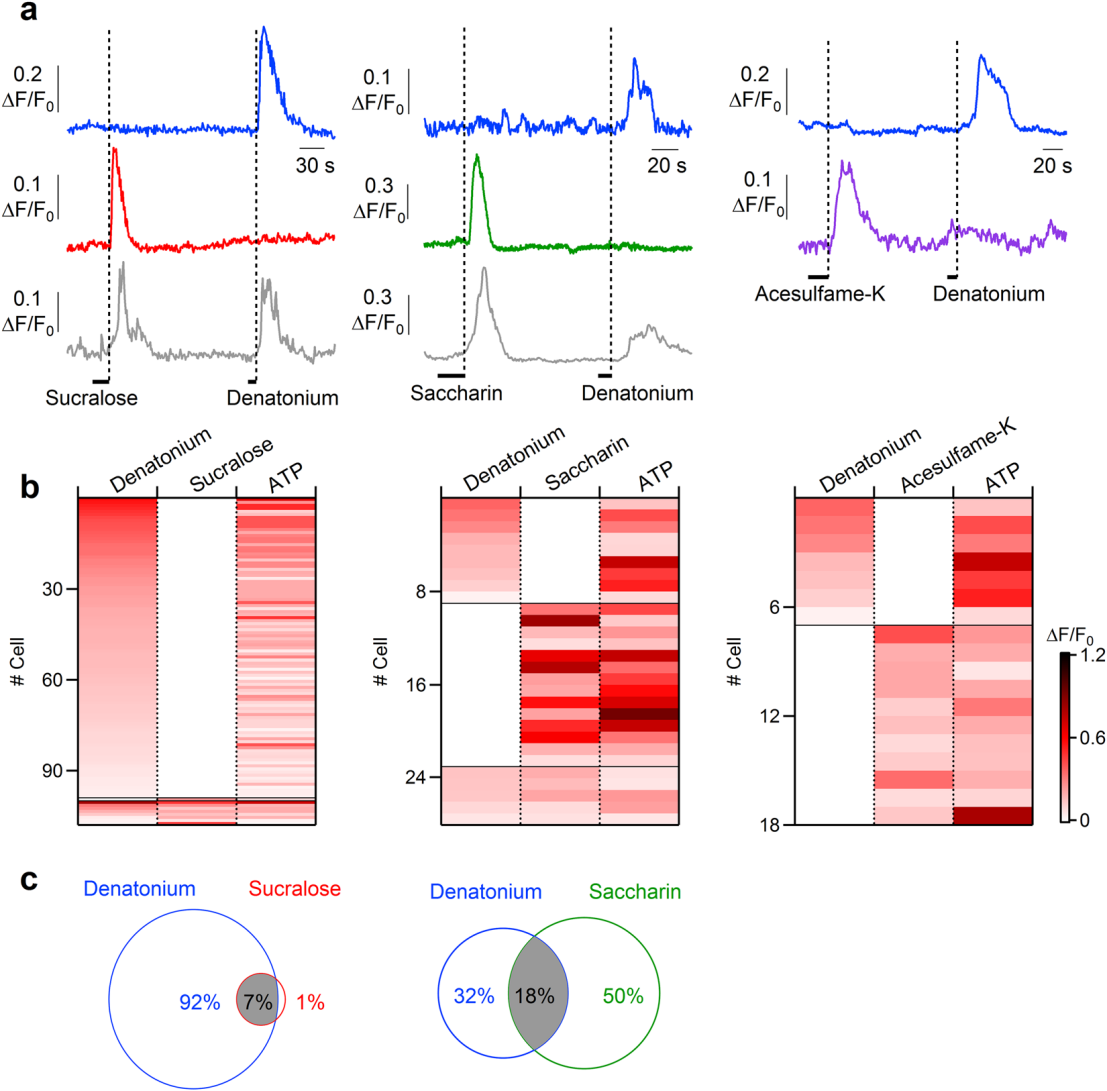
Response profiles of tracheal epithelial cells to bitter tastants and artificial sweeteners. (**a**) Representative calcium transients recorded from epithelial cells stimulated with 5 mM denatonium or artificial sweeteners (20 mM sucralose, 10 mM saccharin and 20 mM acesulfame-K). Each trace represents a different cell. (**b**) Heat map of normalized change in fluorescence intensity following stimulation with the indicated compounds. Thin black lines indicate divisions among cells with different response profiles. Only epithelial cells responsive to one or both of the tested compounds are represented. (**c**) Venn diagram of response overlap for cells that responded to denatonium and/or sucralose, or to denatonium and/or saccharin. Acesulfame-K and denatonium activated distinct populations of epithelial cells.

To investigate the specificity of epithelial cells for tastants, we stimulated the same cells with various combinations of tastants. We first tested an artificial sweetener, followed by denatonium benzoate. The left columns of Fig. 7a–c show the response profile of different epithelial cells responding to sucralose and/or denatonium. Among cells responding to one or both of these two compounds, we found that most of the ATP-responsive cells (from 14 slices) responded only to denatonium (92%, 99 of 108), some responded both to denatonium and sucralose (7%, 8 of 108), and 1 cell of 108 was activated only by sucralose. Similar experiments were performed with other compounds combinations. When saccharin or denatonium were tested (Fig. 7a–c, central columns), most of the ATP-responsive cells (from 11 slices) were activated only by saccharin (50%, 14 of 28), several only by denatonium (32%, 9 of 28), and some both by denatonium and saccharin (18%, 5 of 28). Differently from the previous combinations, stimulation by acesulfame-K or denatonium activated distinct populations of ATP-responsive cells (from 5 slices). Indeed, acesulfame-K induced a Ca^2+^ signal in 61% (11 of 18) and denatonium in 39% of the ATP-responsive cells (7 of 18) responding to one of the two compounds (Fig. 7a–c, right columns).

Altogether, these results show that some bitter tastants and artificial sweeteners produce a dose-dependent increase in intracellular Ca^2+^ in rat tracheal epithelial cells. Moreover, epithelial cells are heterogeneous in their responses to tastants with a subpopulation of tracheal epithelial cells able to respond to both artificial sweeteners (sucralose or saccharin) and bitter (denatonium) tastants.

### Bitter and artificial sweeteners stimulation activate a PLC-mediated Ca^2+^ release from intracellular stores

**Figure 8:**
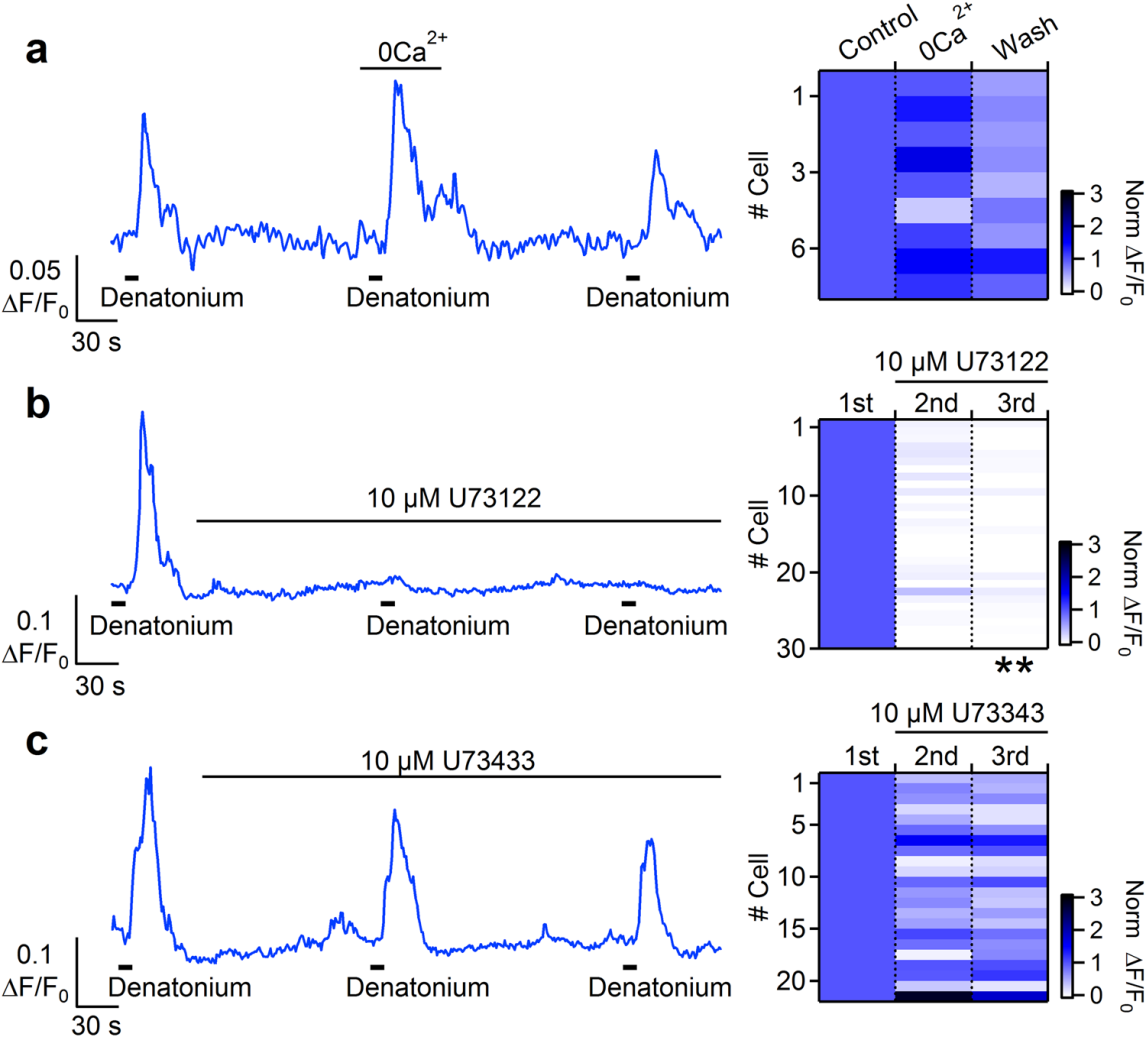
Bitter stimulation activates a PLC mediated Ca^2+^ release from intracellular store in tracheal epithelial cells. (**a**) Representative calcium transients recorded from a cell repeatedly stimulated with 5 mM denatonium in control Ringer’s solution or in the absence of extracellular Ca^2+^. The same experiments were repeated after application of (**b**) 10 µM PLC inhibitor U73122, or (**c**) 10 µM of the inactive analogue U73433 Right panels: heat maps from several cells showing normalized changes in fluorescence intensity following stimulations with denatonium in the conditions indicated in the corresponding left panels. All data were normalized to the peak value obtained from the first stimulation (data from 1–4 slices; **P < 0.01 U-test).

To gain insights into the origin of the Ca^2+^ signal and to determine whether the responses to bitter tastants and artificial sweeteners depended on extracellular Ca^2+^, we stimulated tracheal slices with the different tastants in normal or in Ca^2+^-free Ringer’s solutions. Figure 8a shows that denatonium was still able to induce a significant increase in intracellular Ca^2+^, even if the slice was perfused with Ca^2+^-free Ringer’s solution, suggesting that denatonium evoked a release of Ca^2+^ from intracellular stores. To test if the intracellular Ca^2+^ increase could be mediated by activation of PLCβ2 we applied the PLC inhibitor U73122 to tracheal slices and found an almost complete loss of response to denatonium in all tested cells (Fig. 8b). As control, the application of the inactive analogue U73433 did not abolish denatonium responses (Fig. 8c).

**Figure 9:**
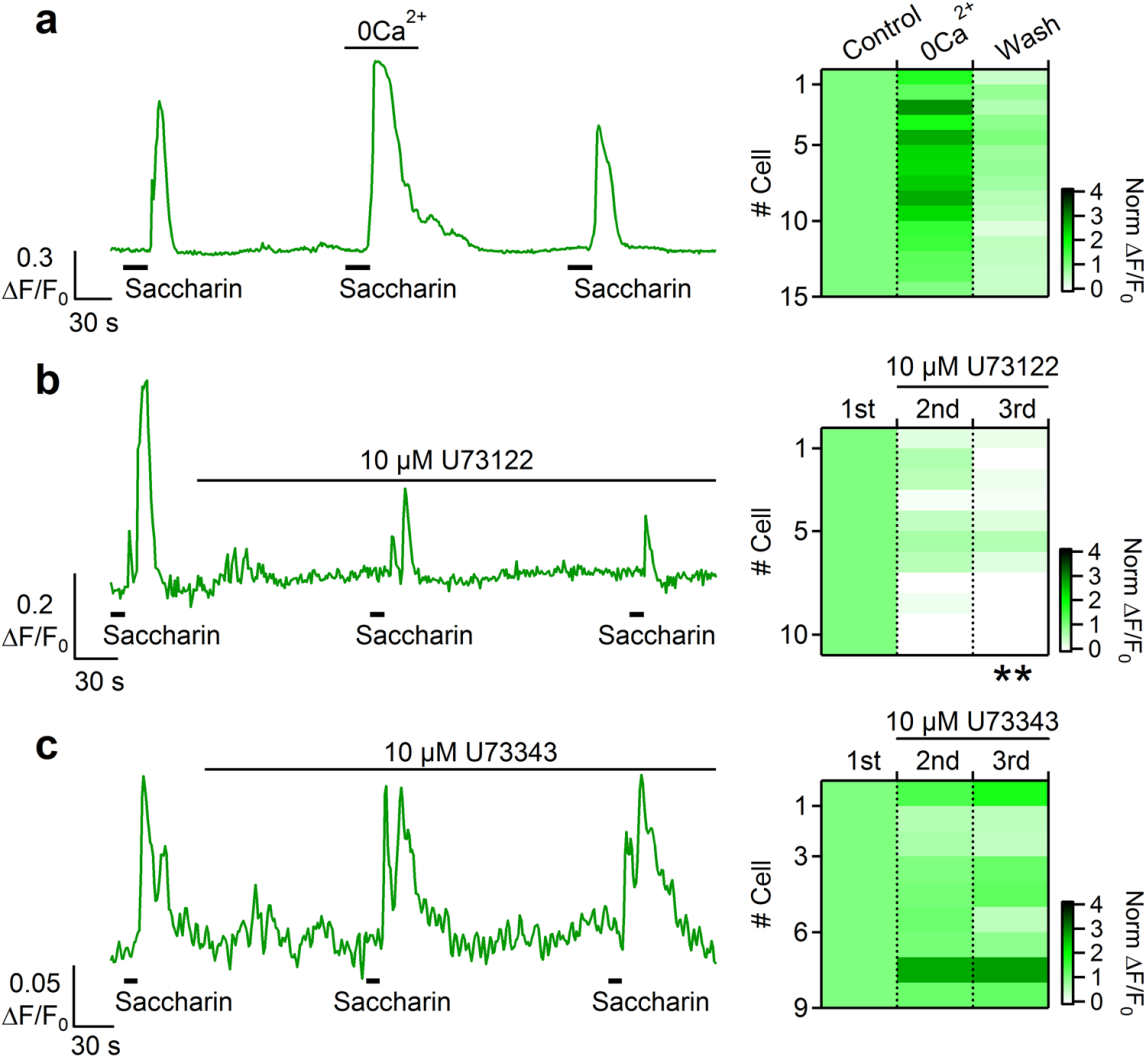
Stimulation with artificial sweeteners activates a PLC mediated Ca^2+^ release from intracellular store in tracheal epithelial cells. (**a**) Representative calcium transient recorded from a cell repeatedly stimulated with 10 mM saccharin in control Ringer’s solution or in the absence of extracellular Ca^2+^. The same experiments were repeated after application of (**b**) 10 µM PLC inhibitor U73122, or (**c**) 10 µM of the inactive analogue U73433. Right panels: heat maps from several cells showing normalized changes in fluorescence intensity following stimulations with denatonium in the conditions indicated in the corresponding left panels. All data were normalized to the peak value obtained from the first stimulation (data from 1–2 slices; **P < 0.01 U-test).

We did the same kind of experiments with saccharin. Similar to the results obtained with denatonium, stimulation with saccharin evoked a Ca^2+^ signal also in the absence of extracellular Ca^2+^, indicating a mobilization from intracellular compartments (Fig. 9a). Moreover, U73122 greatly reduced the rise of Ca^2+^ concentration induced by saccharin stimulation, whereas the inactive analogue U73433 did not decrease responses to saccharin (Fig. 9b,c).

All these data show that in tracheal epithelial cells both bitter tastants and artificial sweeteners activated a PLC-mediated Ca^2+^ release from intracellular stores.

## Discussion

In this study, we have provided the first functional characterization of the responses to various tastants of epithelial cells from acute slices of rat trachea using confocal Ca^2+^ imaging. Our data show that it is possible to record from acute slices of rat trachea, a preparation that preserves the native organization of the epithelial tissue while avoiding possible artifacts due to cell culturing or to cell dissociation. Indeed, individual cells in acute tracheal epithelium slices could be distinguished by their morphology and beating cilia could be observed on ciliated cells (Fig. 1 and Supplementary Video S1). Immunohistochemical analysis with acetylated tubulin further showed the presence of several cilia protruding from the apical surface towards the lumen.

From a functional point of view, we first measured the intracellular Ca^2+^ concentration changes in response to ATP stimulation and found that 70% of epithelial cells in acute tracheal slices respond to ATP with a transient increase of intracellular Ca^2+^ concentration (Fig. 2). Some of these cells are likely to be ciliated cells, as previous data have shown that tracheal ciliated cells from various species respond to ATP stimulation with an increase in intracellular Ca^2+^ concentration^42–44^, but it is also possible that ATP responses originate from additional cell types. Indeed, ATP responses have been measured in studies on brush/microvillous cells from other sources than the rat trachea (where data are not available yet), e.g. nasal SCC^50^, nasal microvillous cells^51^ and urethral brush cells in mice^52^. Moreover, cultures of non-ciliated airway epithelial cells express purinergic receptors and respond to ATP with increase in intracellular calcium^53^. Thus, several cell types may respond to ATP in tracheal slices.

We tested the responsiveness to the bitter tastant denatonium and found that 39% of rat ATP-responsive tracheal epithelial cells responded with a transient intracellular change in Ca^2+^ concentration (Fig. 3). In addition to showing responses of some cells to the bitter tastant denatonium, we have also provided the first direct demonstration that some of these cells respond to artificial sweeteners. Indeed, to the best of our knowledge, sweet tastants have not been previously tested on the tracheal epithelium. We found that sucralose, saccharin and acesulfame-K elicited Ca^2+^ responses respectively in 5%, 26% or 27% of the ATP-responsive rat tracheal epithelial cells and that the responses induced by denatonium and artificial sweeteners were dose-dependent (Figs 6 and 7).

Moreover, addition of the gap junction blocker carbenoxolone did not block the responses to denatonium or saccharin indicating that the activation of tracheal epithelial cells was not mainly due to the spread of Ca^2+^ signaling through gap junctions between neighboring cells (Fig. 5).

Furthermore, we showed that not only some ATP-responsive tracheal epithelial cells respond to artificial sweeteners, but that some of them also respond both to bitter tastants and artificial sweeteners (Fig. 7). Among cells responding to denatonium and/or saccharin, we found that 18% of the ATP-responsive cells were activated by both compounds. When denatonium and sucralose were tested, 7% of the ATP-responsive cells responded to both tastants. It is well known that there are differences in response to sweeteners among species^54^ and that rodents, in particular, show scarce preference to several artificial sweeteners^55^. However, it has been demonstrated that ligand specificities of rat T1R2/T1R3 receptor are similar to those of the human sweet receptor and that both receptors are activated by millimolar concentrations of acesulfame-K, saccharin and sucralose^45^. Moreover, humans, in addition to a sweet taste, also perceive a bitter after-taste with some artificial sweeteners, such as saccharin or acesulfame-K. Indeed, it has been shown that saccharin and acesulfame-K activate not only the sweet, but also the human bitter receptors T2R43 and T2R44^46^, thus accounting for the perceived after-taste of these artificial sweeteners. Although similar data are not yet available for rats, it is possible that some artificial sweeteners also activate rat bitter receptors and therefore we cannot exclude the possibility that saccharin or acesulfame-K activate T2R receptors in rat tracheal epithelial cells. However, at present, sucralose has been shown only to activate human sweet receptors and it is not listed as a bitter compound in the database BitterDB http://bitterdb.agri.huji.ac.il/dbbitter.php ^56,57^. Despite this, we cannot exclude that sucralose also has a bitter off-target in rats.

To test whether the Ca^2+^ transients induced by tastants are due to extracellular Ca^2+^ entry, we performed some experiments in low Ca^2+^ Ringer solution for both bitter tastants and artificial sweeteners (Figs 8 and 9). Similar responses were obtained when extracellular Ca^2+^ was not present, indicating that the Ca^2+^ increase was not due to Ca^2+^ entry. The response was blocked by the PLC inhibitor U73122, but not by the inactive analogue U73433, indicating that the intracellular Ca^2+^ increase originated from a PLC-mediated Ca^2+^ release from intracellular stores.

Based on current knowledge of airway epithelia, at least two epithelial cell types could mediate chemosensation: SCCs/brush cells and ciliated cells. SCCs, including brush cells, are a heterogeneous population, which comprises around 1% of the total tracheal epithelial cells^34^. In rodents, tracheal brush cells are able to respond to bitter–tasting irritants of the luminal microenvironment by releasing acetylcholine which transmits information to sensory nerve endings or to neighboring cells in a paracrine function^31,32,34^. Ciliated cells in cultures obtained from human trachea and bronchi explants have also been shown to directly respond to some bitter stimuli^37,47^. The high percentage of responding cells in our tracheal slices lead us to exclude that we only imaged SCCs, and the persistence of responses after addition of carbenoxolone rules out the possibility that responses were mainly due to the spread of Ca^2+^ waves between neighboring cells. Thus, our results suggest that the observed responses to bitter tastants and artificial sweeteners are mainly due to the activation of tracheal ciliated cells either through binding to taste receptors on these cells, or through a paracrine action via cholinergic transmission induced by tracheal brush cells^31,32,34^. It is likely, in this case, that different types of cells form anatomo-functional units in which the cell cooperation becomes essential.

It is of interest to note that previous studies have reported some differences between responses to denatonium in the lower and upper airways. Indeed, Lee *et al*.^47^ showed that denatonium induced global Ca^2+^ changes in cultures of human bronchial epithelial cells (lower airway) and that dissociated bronchial ciliated epithelial cells responded to denatonium. On the contrary, in cultures of sinonasal epithelial cells (upper airway) the same authors^47^ observed that denatonium induced only some localized Ca^2+^ increases, likely originating from the activation of T2Rs in SCCs and the generation of Ca^2+^ waves in neighboring cells through gap junctions (blocked by carbenoxolone). Moreover, dissociated nasal ciliated epithelial cells did not respond to denatonium, suggesting that T2Rs responsive to denatonium are not expressed in sinonasal ciliated cells, while for example T2R38 is expressed^39^. These results might be explained by a different expression of T2Rs in various cell types of the upper and lower airways.

Additional evidence also points toward involvement of airway ciliated cells in the sensing of the airway lining fluid content, being provided with taste signaling pathway-associated molecules including T1R3 subunit^37,41^. Therefore, we cannot exclude that the detection of the artificial sweeteners may occur by T2R2 or T1R3 receptors (or by additional receptors) expressed in tracheal ciliated cells. Behavioral data have established T1R3 as the primary receptor for the artificial sweeteners showing that mice lacking T1R3 (T1R3 KO mice) have no preferences for artificial sweeteners (i.e. sucralose and acesulfame K) but maintain diminished responses to sugars and umami and unaltered responses to bitter, sour and salty compounds. In addition, T1R3 KO mice lack T1R3 protein expression but preserve that of T1R2^58^. Indeed, although sweet receptors are mainly composed by heterodimers of the T1R2/T1R3 subunits, each of the two subunits T1R2 and T1R3 are capable of binding sweet stimuli^59^ and the presence of sweet sensors composed only by T1R3 (or additional subunits different from T1R2) receptors has been shown in some tissues. Nelson *et al*.^9^ reported the presence in the tongue of cell types expressing only the subunit T1R3 and suggested that T1R3 homomeric receptors, or T1R3 in combination with an additional yet undiscovered receptor, may function as additional sweet sensors. Cells in other tissues have a much lower expression of T1R2 compared to T1R3 indicating that a high percentage of T1R3 is likely to be present as a homomer. For example, homodimers of T1R3 are expressed in mouse pancreatic *β*-cells and their functionality has been assessed by using sucralose, showing that activation of T1R3 by sucralose upregulates insulin secretion^60–62^. It has been reported that mouse adipocytes express functional sweet taste receptor possibly as a T1R3 homomer and stimulation with sucralose or saccharin negatively regulates adipogenesis^63,64^. In rodents, it has been recently demonstrated that T1R3 is expressed in the pulmonary endothelium, where its activation by sucralose protects, *in vitro* and *in vivo*, the endothelium from edemagenic agent-induced barrier disruption^65^.

The role of sweet compounds in the airways is important, especially in terms of its relationship with respiratory infections. Glucose concentration in the airway surface liquid is usually kept low by glucose transporters, restricting the growth of respiratory pathogens^66,67^. However, a high glucose concentration in airway surface liquid is found in patients with viral colds, cystic fibrosis, chronic obstructive pulmonary disease, and asthma, increasing the incidence of respiratory infections^68,69^. Thus, understanding the responses to sweet tastants is physiologically relevant.

A recent study^70^ showed an increased mucociliary clearance in upper and lower airways of guinea pig *in vivo* and in human nasal cell cultures in response to denatonium, saccharin or chloroquine. This study provides an interesting pre-clinical model useful for the study of different upper and lower respiratory diseases and for the evaluation of new therapies to improve mucociliary clearance.

The responses to bitter tastants and artificial sweeteners and the expression of T2Rs and T1Rs in the airways indicate that these receptors may be potential drug targets. Indeed, several studies have suggested a drug target role for human bitter receptors expressed in airways. For example, activation of T2R receptors in smooth muscle cells of the airway causes bronchodilation and it was therefore hypothesized that agonists for these receptors might represent a new class of bronchodilators drugs that are under investigation for asthma and airways obstructive pathology^71–74^.

It is likely that these tastants act through their receptors to activate protective signaling responses in the airways. This might be potentially intriguing for respiratory infections in particular for clinical conditions at risk of developing airways infections (e.g. mechanical ventilated patients, immunodeficiency syndromes, diabetes) because epithelial cells receptors of the airways could be considered a potential target for novel drugs aimed to regulate the glucose level in the airways. Moreover, it is also important to mention that genetic variations of bitter or sweet receptor genes could modify the responses to bitter or sweet substances^75–77^. In the same way, this genetic variability might play a role in susceptibility to respiratory infections^78^. This idea might partially explain the old evidence that there is a genetic basis to respiratory infections^79,80^. Thus, also genetic variability features of sweet receptors should be taken into account for future drug research in airway diseases. Recent studies showed that D-aminoacids products of Staphylococcus bacteria could activate SCC sweet taste receptors and inhibit the bitter receptors mediated signaling^81^. Thus, antagonists for sweet receptors could also be used in the treatment of Staphylococcus mediated infections^77^.

Recent studies have indicated additional roles for sweet taste receptors and glucose transporters, as they seem to be implicated in various disorders of glucose metabolism such as diabetes, obesity and neurodegenerative diseases^82^. For example, we have recently shown^83^ that the T1R3 expression pattern in tracheal ciliated cells was reduced in obese rats and the tracheal epithelium of obese animals showed poorly differentiated cells. This altered epithelial morphology seemed to impair the expression of glucose homeostasis molecules.

In summary, our findings show that bitter tastants and artificial sweeteners elicit intracellular Ca^2+^ increases in ATP-responsive epithelial cells, most likely ciliated cells, of rat acute tracheal slices. The expression of different combinations of bitter and sweet receptors are likely to generate the individual ability of tracheal cells to detect bitter and/or sweet compounds. We speculate that several airway cell types with various chemosensory properties work in concert in an integrated cellular network. Future investigations could unravel their roles in health and in pathological conditions with a possible therapeutic aim. Future research on airway epithelial cells will also contribute to clarify the complicated interaction picture between host and bacteria.

## Materials and Methods

### Preparation of acute tracheal slices

Experiments were performed on neonatal (P5–P7) Wistar rats. All animal procedures were carried out in accordance with the guidelines of the Italian Animal Welfare Act and European Union guidelines on animal research under a protocol approved by the ethic committee of SISSA. Rats were decapitated and the trachea was dissected from the surrounding tissues and transferred to ice-cold Ringer’s solution containing (in mM) 140 NaCl, 5 KCl, 2 CaCl_2_, 1 MgCl_2_, 10 HEPES adjusted at pH 7.4. Trachea lumen was washed by gently pipetting with Ringer’s solution.

To obtain acute tracheal slices, we used the same type of preparation previously developed for acute slices of the vomeronasal organ or olfactory epithelium^84–86^. The trachea was embedded in 3% Type I-A agarose prepared in Ringer’s solution once the solution cooled to 38 °C and coronal slices of 200 µm thickness were cut with a vibratome (Vibratome 1000 Plus Sectioning System) and kept in cold oxygenated Ringer’s solution until use.

### Immunohistochemistry

Immunohistochemistry was performed on acute tracheal slices just after sectioning or after Ca^2+^-imaging recordings. Slices were fixed with 4% paraformaldehyde in PBS (Phosphate Buffered Saline), for 20 minutes, incubated with 1% SDS (Sodium Dodecyl Sulfate) in PBS for 15 minutes for antigen retrieval, and then transferred in blocking solution containing 2% fetal bovine serum in TPBS (PBS +0.02% Tween-20) for 1 hour. Slices were incubated overnight at 4 °C with anti-acetylated tubulin monoclonal antibody (Sigma, T7451) diluted 1:1500 in blocking solution, and subsequently incubated for 1 hour at room temperature with AlexaFluor 488 conjugated goat-anti mouse IgG antibody (Thermo Fisher, diluted 1:500 in TPBS). Finally, slices were stained with DAPI (0.1 µg/ml) for 30 minutes and mounted with Vectashield (Vector Laboratories). Images were collected with a Nikon C1 confocal microscope using NIS Element software (Nikon) at 1024 × 1024 pixel resolution and analyzed with ImageJ 1.51 s (NIH).

### Confocal calcium imaging

Slices were loaded with 20 µM Cal-520AM (Santa Cruz Biotechnologies) for 90 minutes at room temperature. To help dye uptake, Pluronic F-127 was added at final concentration of 0.2 mg/ml. Moreover, 100 µM sulfobromophtalein (BSP) was added to prevent the indicator extrusion by organic anion transporter^87^. After wash, the slices were kept in Ringer’s solution until use. Stock solution of Cal-520AM was prepared in dimethyl sulfoxide (DMSO) at 2 mM and stored at −20 °C. Pluronic F-127 was weekly dissolved in DMSO at 200 mg/ml concentration. BSP was directly prepared in Ringer’s solution at working concentration on the day of the experiment.

Slices were placed in a laminar flow chamber (Warner Instruments) and continuously perfused with Ringer’s solution at the rate of about 1 ml/min. A gravity-driven multivalve perfusion system (Automate Scientific) was used to deliver the stimuli.

An inverted Nikon C1 confocal microscope was used for data acquisition with 40X oil-immersion objective (NA 1.3) through the NIS Element software (Nikon). Cal-520 fluorescence was excited using a krypton-argon ion laser with a total power of 15 mW. To reduce dye bleaching and photodamage, only 1–4% of the laser power was used. Fluorescence emission was band-passed at 515 nm with 30 nm of bandwidth. Data were acquired at 1–2 Hz with 512 × 512 or 256 × 256 pixel resolution. Recordings were obtained 50–100 µm below the slice surface to avoid damaged cells.

Ca^2+^-free solution was the same as Ringer’s with the omission of 2 mM CaCl_2_ and the addition of 5 mM EGTA. Stock solutions of U73122 and U73343 were prepared in DMSO at 1 mM and stored at −20 °C. Stock solutions of denatonium benzoate, ATP, and sucralose were prepared in Ringer’s solution at 100 mM, 300 mM, 500 mM, respectively and stored at −20 °C. Acesulfame-K and saccharin were dissolved in Ringer’s solution at working concentration on the day of the experiment.

Chemicals were purchased form Sigma-Aldrich, unless otherwise stated.

### Whole-cell recordings from supporting cells of the olfactory epithelium

Whole-cell recordings from supporting cells were obtained from coronal slices of the olfactory epithelium obtained from the nose of P0-P4 C57BL/6 mice. The nose was dissected en bloc and embedded in 3% Type I-A agarose prepared in Ringer’s solution. Coronal slices of 300 µm thickness were cut with a vibratome as described for tracheal slices, transferred to a recording chamber placed on an upright microscope (BX51WI; Olympus) and continuously perfused with oxygenated Ringer’s solution. Infrared differential contrast optics with a 40X water-immersion objective and a 2X auxiliary lens were used to visualize the slices. Olfactory supporting cells were identified by their morphology. The pipette solution contained (in mM): 140 CsCl, 10 HEDTA, and 10 HEPES, adjusted to pH 7.2 with CsOH. Fluorescein (10 μg/ml) was also added to the pipette solution to have a fluorescence image of the cell under blue light. Patch pipettes were pulled from borosilicate capillaries (WPI) with a Narishige PC-10 puller and had resistances of 3–5 MΩ when filled with the intracellular solution. Whole-cell voltage-clamp recordings were obtained using a MultiClamp 700B amplifier controlled by Clampex 10.6 via a Digidata 1550B (Molecular Devices). Data were low-pass filtered at 2 kHz and sampled at 10 kHz. The input resistance of olfactory supporting cells was calculated by measuring current in response to a −10 mV voltage step of 10 ms duration from the holding potential of −80 mV. Carbenoxolone (Sigma) was prepared in Ringer’s solution at 5 mM, stored at −20 °C, and dissolved in Ringer’s solution at 100 μM on the day of the experiment. IGOR Pro software (WaveMetrics) was used for data analysis.

### Imaging data analysis and statistics

Changes in fluorescence were measured in regions of interest (ROIs) drawn around a single tracheal epithelial cell using ImageJ 1.51 s (NIH). Data are presented as normalized fluorescence changes, ΔF/F_0_ = (F(t) − F_0_)/F_0_, where F_0_ was the average of fluorescence intensity before the application of the first stimulus and F(t) was the fluorescence amplitude at time t. Further analysis and figures were made with IgorPro 6.3.7.2 (Wavemetrics). In some experiments, the reduction of fluorescence signal due to photobleaching was mathematically corrected using the exponential decay observed in non-responding cells^88^. We considered a cell responsive if: (i) after stimulation ΔF/F_0_ was higher than the average of the prestimulus (15 s time window) plus three standard deviation for at least 5 seconds, (ii) there was no response to Ringer’s solution application, and (iii) there was no spontaneous activity.

Since some data were not normally distributed (Shapiro-Wilk’s or Jarque-Bera’s tests), we used the One sample sign test or the Wilcoxon-Mann-Whitney’s test (U-test) to determine the statistical significance of the results (Figs 6, 7 and 8). The statistical differences in the frequencies of responding cells were tested with the chi-squared test (χ^2^-test, Figs 5 and S2). P values < 0.05 were considered statistically significant.

## Supplementary information

Supporting Info

Supporting Video 1

## Data Availability

All datasets in this manuscript are available from the corresponding author upon request.

## References

[1] Carleton A, Accolla R, Simon SA (2010). Coding in the mammalian gustatory system. Trends Neurosci..

[2] Avau B, Depoortere I (2016). The bitter truth about bitter taste receptors: beyond sensing bitter in the oral cavity. Acta Physiol. Oxf. Engl..

[3] Herrera Moro Chao D (2016). Impact of obesity on taste receptor expression in extra-oral tissues: emphasis on hypothalamus and brainstem. Sci. Rep..

[4] Laffitte A, Neiers F, Briand L (2014). Functional roles of the sweet taste receptor in oral and extraoral tissues. Curr. Opin. Clin. Nutr. Metab. Care.

[5] Yamamoto K, Ishimaru Y (2013). Oral and extra-oral taste perception. Semin. Cell Dev. Biol..

[6] Adler E (2000). A novel family of mammalian taste receptors. Cell.

[7] Chandrashekar J (2000). T2Rs function as bitter taste receptors. Cell.

[8] Matsunami H, Montmayeur JP, Buck LB (2000). A family of candidate taste receptors in human and mouse. Nature.

[9] Nelson G (2001). Mammalian sweet taste receptors. Cell.

[10] Zhao GQ (2003). The receptors for mammalian sweet and umami taste. Cell.

[11] Hepler JR, Gilman AG (1992). G proteins. Trends Biochem. Sci..

[12] McLaughlin SK, McKinnon PJ, Margolskee RF (1992). Gustducin is a taste-cell-specific G protein closely related to the transducins. Nature.

[13] Zhang Y (2003). Coding of sweet, bitter, and umami tastes: different receptor cells sharing similar signaling pathways. Cell.

[14] Hisatsune C (2007). Abnormal taste perception in mice lacking the type 3 inositol 1,4,5-trisphosphate receptor. J. Biol. Chem..

[15] Tordoff MG, Ellis HT (2013). Taste dysfunction in BTBR mice due to a mutation of Itpr3, the inositol triphosphate receptor 3 gene. Physiol. Genomics.

[16] Dutta Banik D, Martin LE, Freichel M, Torregrossa A-M, Medler KF (2018). TRPM4 and TRPM5 are both required for normal signaling in taste receptor cells. Proc. Natl. Acad. Sci. USA.

[17] Huang YA, Roper SD (2010). Intracellular Ca(2+) and TRPM5-mediated membrane depolarization produce ATP secretion from taste receptor cells. J. Physiol..

[18] Ma Z (2018). CALHM3 Is Essential for Rapid Ion Channel-Mediated Purinergic Neurotransmission of GPCR-Mediated Tastes. Neuron.

[19] Pérez CA (2002). A transient receptor potential channel expressed in taste receptor cells. Nat. Neurosci..

[20] Taruno A (2013). CALHM1 ion channel mediates purinergic neurotransmission of sweet, bitter and umami tastes. Nature.

[21] Zhang Z, Zhao Z, Margolskee R, Liman E (2007). The transduction channel TRPM5 is gated by intracellular calcium in taste cells. J. Neurosci. Off. J. Soc. Neurosci..

[22] Lu P, Zhang C-H, Lifshitz LM, ZhuGe R (2017). Extraoral bitter taste receptors in health and disease. J. Gen. Physiol..

[23] Finger TE, Kinnamon SC (2011). Taste isn’t just for taste buds anymore. F1000 Biol. Rep..

[24] Kinnamon SC (2012). Taste receptor signalling - from tongues to lungs. Acta Physiol. Oxf. Engl..

[25] Sbarbati A, Bramanti P, Benati D, Merigo F (2010). The diffuse chemosensory system: exploring the iceberg toward the definition of functional roles. Prog. Neurobiol..

[26] Sbarbati A, Osculati F (2005). A new fate for old cells: brush cells and related elements. J. Anat..

[27] Sbarbati A, Osculati F (2005). The taste cell-related diffuse chemosensory system. Prog. Neurobiol..

[28] Merigo F, Benati D, Tizzano M, Osculati F, Sbarbati A (2005). alpha-Gustducin immunoreactivity in the airways. Cell Tissue Res..

[29] Tizzano M, Merigo F, Sbarbati A (2006). Evidence of solitary chemosensory cells in a large mammal: the diffuse chemosensory system in Bos taurus airways. J. Anat..

[30] Tizzano M, Cristofoletti M, Sbarbati A, Finger TE (2011). Expression of taste receptors in solitary chemosensory cells of rodent airways. BMC Pulm. Med..

[31] Krasteva G (2011). Cholinergic chemosensory cells in the trachea regulate breathing. Proc. Natl. Acad. Sci. USA.

[32] Krasteva G, Canning BJ, Papadakis T, Kummer W (2012). Cholinergic brush cells in the trachea mediate respiratory responses to quorum sensing molecules. Life Sci..

[33] Kummer W, Krasteva-Christ G (2014). Non-neuronal cholinergic airway epithelium biology. Curr. Opin. Pharmacol..

[34] Saunders CJ, Reynolds SD, Finger TE (2013). Chemosensory brush cells of the trachea. A stable population in a dynamic epithelium. Am. J. Respir. Cell Mol. Biol..

[35] Bustamante-Marin, X. M. & Ostrowski, L. E. Cilia and Mucociliary Clearance. *Cold Spring Harb*. *Perspect*. *Biol*. **9** (2017). 10.1101/cshperspect.a028241 PMC5378048 27864314

[36] Fliegauf M, Benzing T, Omran H (2007). When cilia go bad: cilia defects and ciliopathies. Nat. Rev. Mol. Cell Biol..

[37] Shah AS, Ben-Shahar Y, Moninger TO, Kline JN, Welsh MJ (2009). Motile cilia of human airway epithelia are chemosensory. Science.

[38] Hariri BM (2017). Flavones modulate respiratory epithelial innate immunity: Anti-inflammatory effects and activation of the T2R14 receptor. J. Biol. Chem..

[39] Lee RJ (2012). T2R38 taste receptor polymorphisms underlie susceptibility to upper respiratory infection. J. Clin. Invest..

[40] Yan CH (2017). Nitric oxide production is stimulated by bitter taste receptors ubiquitously expressed in the sinonasal cavity. Am. J. Rhinol. Allergy.

[41] Merigo F (2012). Glucose transporter/T1R3-expressing cells in rat tracheal epithelium. J. Anat..

[42] Korngreen A, Priel Z (1996). Purinergic stimulation of rabbit ciliated airway epithelia: control by multiple calcium sources. J. Physiol..

[43] Li W-E (2012). Methods to measure and analyze ciliary beat activity: Ca^2+^ influx-mediated cilia mechanosensitivity. Pflugers Arch..

[44] Ma W (2006). Pore properties and pharmacological features of the P2X receptor channel in airway ciliated cells. J. Physiol..

[45] Li X (2002). Human receptors for sweet and umami taste. Proc. Natl. Acad. Sci. USA.

[46] Kuhn C (2004). Bitter taste receptors for saccharin and acesulfame K. J. Neurosci. Off. J. Soc. Neurosci..

[47] Lee RJ (2014). Bitter and sweet taste receptors regulate human upper respiratory innate immunity. J. Clin. Invest..

[48] Rozental R, Srinivas M, Spray DC (2001). How to close a gap junction channel. Efficacies and potencies of uncoupling agents. Methods Mol. Biol. Clifton NJ.

[49] Vogalis F, Hegg CC, Lucero MT (2005). Ionic conductances in sustentacular cells of the mouse olfactory epithelium. J. Physiol..

[50] Gulbransen BD, Clapp TR, Finger TE, Kinnamon SC (2008). Nasal solitary chemoreceptor cell responses to bitter and trigeminal stimulants *in vitro*. J. Neurophysiol..

[51] Fu, Z., Ogura, T., Luo, W. & Lin, W. ATP and Odor Mixture Activate TRPM5-Expressing Microvillous Cells and Potentially Induce Acetylcholine Release to Enhance Supporting Cell Endocytosis in Mouse Main Olfactory Epithelium. *Front*. *Cell*. *Neurosci*. **12** (2018). 10.3389/fncel.2018.00071 PMC5869921 29615870

[52] Kandel C (2018). ENaC in Cholinergic Brush Cells. Front. Cell Dev. Biol..

[53] Zsembery A (2004). Extracellular zinc and ATP restore chloride secretion across cystic fibrosis airway epithelia by triggering calcium entry. J. Biol. Chem..

[54] Hellekant G, Danilova V (1996). Species differences toward sweeteners. Food Chem..

[55] Danilova V, Hellekant G, Tinti JM, Nofre C (1998). Gustatory responses of the hamster Mesocricetus auratus to various compounds considered sweet by humans. J. Neurophysiol..

[56] Dagan-Wiener A (2017). Bitter or not? BitterPredict, a tool for predicting taste from chemical structure. Sci. Rep..

[57] Wiener A, Shudler M, Levit A, Niv MY (2012). BitterDB: a database of bitter compounds. Nucleic Acids Res..

[58] Damak S (2003). Detection of sweet and umami taste in the absence of taste receptor T1r3. Science.

[59] Nie Y, Vigues S, Hobbs JR, Conn GL, Munger SD (2005). Distinct contributions of T1R2 and T1R3 taste receptor subunits to the detection of sweet stimuli. Curr. Biol. CB.

[60] Kojima I (2015). Glucose-Sensing Receptor T1R3: A New Signaling Receptor Activated by Glucose in Pancreatic β-Cells. Biol. Pharm. Bull..

[61] Medina A (2014). Expression of the glucose-sensing receptor T1R3 in pancreatic islet: changes in the expression levels in various nutritional and metabolic states. Endocr. J..

[62] Nakagawa Y, Ohtsu Y, Nagasawa M, Shibata H, Kojima I (2014). Glucose promotes its own metabolism by acting on the cell-surface glucose-sensing receptor T1R3. Endocr. J..

[63] Masubuchi Y (2013). A novel regulatory function of sweet taste-sensing receptor in adipogenic differentiation of 3T3-L1 cells. PloS One.

[64] Masubuchi Y (2017). T1R3 homomeric sweet taste receptor regulates adipogenesis through Gαs-mediated microtubules disassembly and Rho activation in 3T3-L1 cells. PloS One.

[65] Harrington EO, Vang A, Braza J, Shil A, Chichger H (2018). Activation of the sweet taste receptor, T1R3, by the artificial sweetener sucralose regulates the pulmonary endothelium. Am. J. Physiol. Lung Cell. Mol. Physiol..

[66] Pezzulo AA (2011). Glucose depletion in the airway surface liquid is essential for sterility of the airways. PloS One.

[67] Philips BJ, Meguer J-X, Redman J, Baker EH (2003). Factors determining the appearance of glucose in upper and lower respiratory tract secretions. Intensive Care Med..

[68] Baker EH (2007). Hyperglycemia and cystic fibrosis alter respiratory fluid glucose concentrations estimated by breath condensate analysis. J. Appl. Physiol. Bethesda Md 1985.

[69] Garnett JP (2012). Proinflammatory mediators disrupt glucose homeostasis in airway surface liquid. J. Immunol. Baltim. Md 1950.

[70] Ortiz JL (2016). Evaluation of Mucociliary Clearance by Three Dimension Micro-CT-SPECT in Guinea Pig: Role of Bitter Taste Agonists. PloS One.

[71] An SS (2012). TAS2R activation promotes airway smooth muscle relaxation despite β(2)-adrenergic receptor tachyphylaxis. Am. J. Physiol. Lung Cell. Mol. Physiol..

[72] Deshpande DA (2010). Bitter taste receptors on airway smooth muscle bronchodilate by localized calcium signaling and reverse obstruction. Nat. Med..

[73] Liggett, S. B. Bitter taste receptors in the wrong place: novel airway smooth muscle targets for treating asthma. *Trans*. *Am*. *Clin*. *Climatol*. *Assoc*. **125**, 64–74; discussion 74–75 (2014). PMC4112707 25125719

[74] Robinett KS, Deshpande DA, Malone MM, Liggett SB (2011). Agonist-promoted homologous desensitization of human airway smooth muscle bitter taste receptors. Am. J. Respir. Cell Mol. Biol..

[75] Bachmanov AA (2014). Genetics of taste receptors. Curr. Pharm. Des..

[76] Fushan AA, Simons CT, Slack JP, Manichaikul A, Drayna D (2009). Allelic polymorphism within the TAS1R3 promoter is associated with human taste sensitivity to sucrose. Curr. Biol. CB.

[77] Triantafillou V, Workman AD, Kohanski MA, Cohen NA (2018). Taste Receptor Polymorphisms and Immune Response: A Review of Receptor Genotypic-Phenotypic Variations and Their Relevance to Chronic Rhinosinusitis. Front. Cell. Infect. Microbiol..

[78] Lee RJ, Cohen NA (2014). Bitter and sweet taste receptors in the respiratory epithelium in health and disease. J. Mol. Med. Berl. Ger..

[79] Greisner WA, Settipane GA (1996). Hereditary factor for nasal polyps. Allergy Asthma Proc..

[80] Hamilos DL (2007). Approach to the evaluation and medical management of chronic rhinosinusitis. Clin. Allergy Immunol..

[81] Lee Robert J., Hariri Benjamin M., McMahon Derek B., Chen Bei, Doghramji Laurel, Adappa Nithin D., Palmer James N., Kennedy David W., Jiang Peihua, Margolskee Robert F., Cohen Noam A. (2017). Bacterial d-amino acids suppress sinonasal innate immunity through sweet taste receptors in solitary chemosensory cells. Science Signaling.

[82] Welcome Menizibeya O., Mastorakis Nikos E. (2018). Emerging Concepts in Brain Glucose Metabolic Functions: From Glucose Sensing to How the Sweet Taste of Glucose Regulates Its Own Metabolism in Astrocytes and Neurons. NeuroMolecular Medicine.

[83] Merigo F, Boschi F, Lasconi C, Benati D, Sbarbati A (2016). Molecules implicated in glucose homeostasis are differentially expressed in the trachea of lean and obese Zucker rats. Eur. J. Histochem. EJH.

[84] Dibattista M, Mazzatenta A, Grassi F, Tirindelli R, Menini A (2008). Hyperpolarization-activated cyclic nucleotide-gated channels in mouse vomeronasal sensory neurons. J. Neurophysiol..

[85] Pietra G, Dibattista M, Menini A, Reisert J, Boccaccio A (2016). The Ca^2+^ -activated Cl^−^ channel TMEM16B regulates action potential firing and axonal targeting in olfactory sensory neurons. J. Gen. Physiol..

[86] Shimazaki R (2006). Electrophysiological properties and modeling of murine vomeronasal sensory neurons in acute slice preparations. Chem. Senses.

[87] Delmotte P, Sanderson MJ (2006). Ciliary beat frequency is maintained at a maximal rate in the small airways of mouse lung slices. Am. J. Respir. Cell Mol. Biol..

[88] Thomas D (2000). A comparison of fluorescent Ca^2+^ indicator properties and their use in measuring elementary and global Ca^2+^ signals. Cell Calcium.

