# Supplementary material for: Bitter tastants and artificial sweeteners activate a subset of epithelial cells in acute tissue slices of the rat trachea
Source: Sci Rep. 2019 Jun 20;9:8834. doi: 10.1038/s41598-019-45456-w (PMC6586933; doi:10.1038/s41598-019-45456-w)
Supplement: Supplementary file 1 — Supporting Info [file 41598_2019_45456_MOESM1_ESM.pdf]

Bitter tastants and artificial sweeteners activate a subset of epithelial cells in acute tissue slices of the rat trachea

Chiara Lasconi[1*], Simone Pifferi[2*#], Andres Hernandez-Clavijo[2*], Flavia Merigo[1], Maria Paola Cecchini[1#], Kevin Y. Gonzalez-Velandia[2], Emilio Agostinelli[2], Andrea Sbarbati[1], Anna Menini[2#]

[1] Department of Neurosciences, Biomedicine and Movement Sciences, Anatomy and Histology Section, University of Verona, School of Medicine, Verona, Italy

[2] Neurobiology Group, SISSA, International School for Advanced Studies, Trieste, Italy

* These authors equally contributed to this work

# Corresponding authors

Correspondence to spifferi@sissa.it or mariapaola.cecchini@univr.it

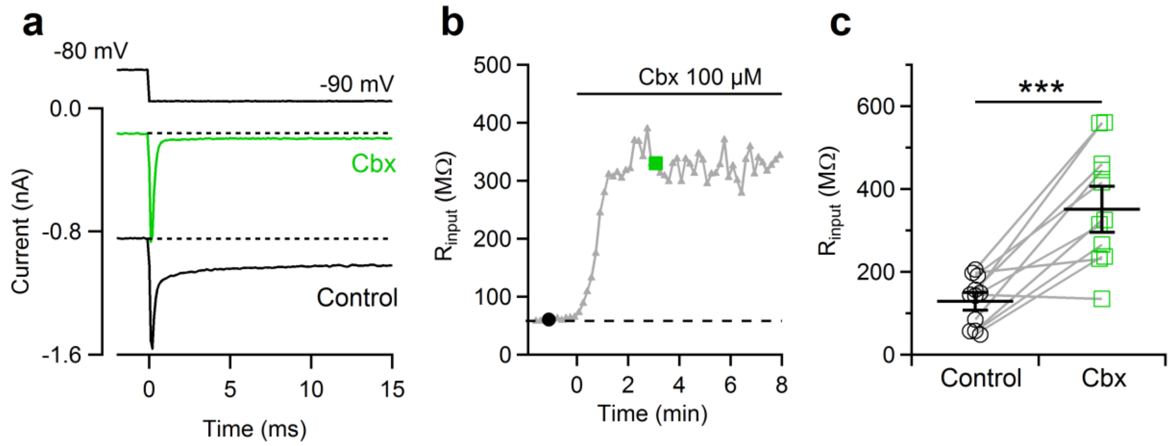

Supplementary Figure 1

(a) Control experiments for the block of gap junctions by carbenoxolone. Representative whole-cell currents recorded from a supporting cell from a slice of the olfactory epithelium in response to the voltage protocol indicated at the top of the panel. The black trace was recorded during Ringer perfusion (control; black) and the green trace after 3 minutes of 100 µM carbenoxolone application (Cbx; green). (b) Time course of the increase of the input resistance during carbenoxolone (Cbx) application. Black and green dots represent the values obtained from the traces in (a). (c) Scatter dot plot showing the input resistance values before (black circles) and after 3 minutes of Cbx application (green square, n = 11 cells). Horizontal lines represent the mean and error bars the SEM (*** $p<0.001$ U-test).

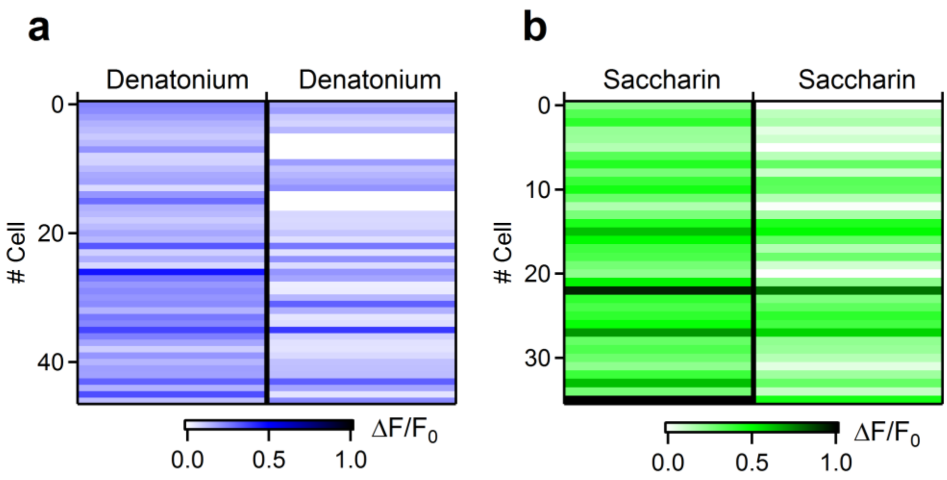

Supplementary Figure 2
Heat maps of normalized change in fluorescence intensity following stimulation with 5 mM denatonium (a) or 20 mM saccharin (b) before (left column) and after (right column) 5 min of perfusion with Ringer’s solution.

Supplementary Video S1.
Calcium fluorescence in a rat tracheal slice loaded with Cal520-AM and stimulated with 30 µM ATP for 10 s (left) and bright-field video (right). Arrows indicate two ciliated cells with visible changes in fluorescence in the cilia.
